# APOBEC3C Tandem Domain Proteins Create Super Restriction Factors against HIV-1

**DOI:** 10.1128/mBio.00737-20

**Published:** 2020-04-28

**Authors:** Mollie M. McDonnell, Kate H. D. Crawford, Adam S. Dingens, Jesse D. Bloom, Michael Emerman

**Affiliations:** a Molecular and Cellular Biology Graduate Program, University of Washington, Seattle, Washington, USA; b Division of Human Biology, Fred Hutchinson Cancer Research Center, Seattle, Washington, USA; c Division of Basic Sciences, Fred Hutchinson Cancer Research Center, Seattle, Washington, USA; d UW Genome Sciences Graduate Program, Seattle, Washington, USA; e UW Medical Scientist Training Program, Seattle, Washington, USA; f Howard Hughes Medical Institute, Seattle, Washington, USA; Columbia University/ HHMI

**Keywords:** APOBEC3C, HIV-1, restriction factor, Vif, innate immunity

## Abstract

Humans encode proteins, called restriction factors, that inhibit replication of viruses such as HIV-1. The members of one family of antiviral proteins, *apolipoprotein B mRNA-editing enzyme catalytic polypeptide-like 3* (APOBEC3; shortened here to A3), act by deaminating cytidines to uridines during the reverse transcription reaction of HIV-1. The *A3* locus encodes seven genes, named *A3A* to *A3H*. These genes have either one or two cytidine deaminase domains, and several of these A3s potently restrict HIV-1. A3C, which has only a single cytidine deaminase domain, however, inhibits HIV-1 only very weakly. We tested novel double domain protein combinations by genetically linking two *A3C* genes to make a synthetic tandem domain protein. This protein created a “super restriction factor” that had more potent antiviral activity than the native A3C protein, which correlated with increased packaging into virions. Furthermore, disabling one of the active sites of the synthetic tandem domain protein resulted in an even greater increase in the antiviral activity—recapitulating a similar evolution seen in A3F and A3G (double domain A3s that use only a single catalytically active deaminase domain). These A3C tandem domain proteins do not have an increase in mutational activity but instead inhibit formation of reverse transcription products, which correlates with their ability to form large higher-order complexes in cells. Finally, the A3C-A3C super restriction factor largely escaped antagonism by the HIV-1 viral protein Vif.

## Abstract (precis)

As a part of the innate immune system, humans encode proteins that inhibit viruses such as HIV-1. These broadly acting antiviral proteins do not protect humans from viral infections because viruses encode proteins that antagonize the host antiviral proteins to evade the innate immune system. One such example of a host antiviral protein is APOBEC3C (A3C), which weakly inhibits HIV-1. Here, we show that we can improve the antiviral activity of A3C by duplicating the DNA sequence to create a synthetic tandem domain and, furthermore, that the proteins thus generated are relatively resistant to the viral antagonist Vif. Together, these data give insights about how nature has evolved a defense against viral pathogens such as HIV.

## INTRODUCTION

The *apolipoprotein B mRNA-editing enzyme catalytic polypeptide-like 3* (*APOBEC3*; shortened here to *A3*) gene locus in primates encodes cytidine deaminase proteins that inhibit endogenous retroelements, such as LINE-1, and retroviruses, such as HIV-1, among other viruses, including hepatitis B virus, human papillomavirus, and some herpesviruses (1–4). This *A3* gene locus has expanded in primates to give rise to the seven members of the *A3* family, named *A3A* to *A3H* (5). For A3 proteins to inhibit HIV-1 replication, they must be packaged into budding virions and brought to the target cell, where they extensively deaminate cytidines on the negative strand of single-stranded DNA (ssDNA) to uridines during reverse transcription (RT) (1, 2). The resultant G-to-A hypermutation on the positive strand renders the provirus nonfunctional. The most potent naturally found A3, A3G, can mutate up to 10% of the guanines on a single provirus (6). In addition to extensive hypermutation, some A3s can also inhibit reverse transcription via deamination-independent mechanisms as demonstrated by the presence of truncated cDNA products in the presence of A3s (1, 7–10). Recent studies of A3G showed that this nonenzymatic mechanism functions by binding and sterically hindering the enzymatic activity of reverse transcriptase (11).

There are four human A3s that have endogenous antiviral activity against HIV-1 in T cells, A3D, A3F, A3G, and A3H, with A3G being the most potent (12). In order to replicate in the presence of these A3s, HIV-1 and other lentiviruses encode a protein, Vif, that targets A3s for proteasomal degradation. Vif has evolved three separate interfaces in order to degrade A3s: one binding A3G, another A3H, and a third able to recruit A3C/A3D/A3F (2). Moreover, Vif also binds to the host factor CBF-β to help recruit an E3 ubiquitin ligase complex composed of CUL5, ELOB, ELOC, RBX2, and ARIH2 proteins that mediate polyubiquitination and rapid degradation of the A3s through the proteasome (13, 14).

In addition to the multiple *A3* genes in humans, there is additional diversity in the *A3* locus in the form of human polymorphisms that affect the antiviral activity against HIV-1. For instance, there are at least twelve haplotypes of A3H that vary in their ability to restrict HIV-1 (15, 16). In addition, the most common form of human A3C, which codes for a serine at position 188, has little, if any, activity against HIV-1. However, about 10% of African individuals carry a polymorphism in A3C that encodes an isoleucine at position 188. This one amino acid change from a serine to an isoleucine is correlated with increased antiviral activity against HIV-1 resulting from an increased ability of A3C to form dimers and increased cytidine deaminase activity *in vitro* (17). Furthermore, chimpanzee and gorilla A3Cs have amino acids at position 115 that differ from those in humans and introducing these amino acids into human A3C also increases dimerization and cytidine deaminase activity (18). Nonetheless, even with these mutations, A3C is less active against HIV-1 than many of the other A3 proteins (19).

Each *A3* gene can be classified according to the presence of a zinc-coordinating domain motif: A3Z1, A3Z2, or A3Z3 (20). The evolutionary history of the *A3* locus is characterized by duplication, recombination, and deletion events; in the last 50 million years, three duplication events occurred in the A3Z1 and A3Z2 subfamilies, but not in the A3Z3 subfamily (5). These fusion and recombination events gave rise to the seven *A3* genes found in primates that include the three single-deaminase-domain *A3* genes (*A3A*, *A3C*, and *A3H*) and four double-deaminase-domain *A3* genes (*A3B*, *A3D*, *A3F*, and *A3G*).

Super restriction factors are defined as evolution-guided variants of natural restriction factors with improved properties based on previous work done on the restriction factor MXA (21). Super restriction factors provide a unique tool to study how restriction factors work and the evolutionary compromises for paths that have yet to be sampled in nature. Because the most active A3s are double domain proteins, we examined the hypothesis that super restrictor factors could be made by duplicating the poorly active single domain A3C protein into a synthetic tandem domain protein. We did this for both the common form of human A3C (A3C_S188_) and the more active variant of A3C (A3C_I188_). Remarkably, we found that all A3C tandem domain variants had greater antiviral activity than their single domain counterparts. We found that the tandem deaminase A3C variants are packaged into virions at higher levels than their single-domain counterparts, likely explaining the majority of the increase in antiviral activity. In the natural double domain APOBEC3 proteins, only the C-terminal cytidine deaminase active sites are used for hypermutation (22, 23). Here, we show that mutation of the cytidine deaminase active site in the C-terminal domain of A3C_S188_-A3C_S188_ results in even greater antiviral activity than that seen with the same protein with two active sites. This increase in antiviral activity is correlated with elevated packaging into virions and is largely independent of increased mutational load. Instead, there were smaller amounts of reverse transcriptase products in cells infected with virus produced in the presence of these A3C tandem domain proteins. This inhibition of late reverse transcription products is also correlated with the formation of large higher-order complexes in the A3C-A3C variants compared to their single domain counterparts. Finally, we show that the A3C tandem domain proteins are largely resistant to antagonism by HIV-1 Vif. These A3C-A3C super restriction factors provide a unique tool to understand evolutionary paths of antiviral genes and their antagonism by viral proteins.

## RESULTS

### Synthetic tandem domains of A3C have increased antiviral activity and are better packaged into virions.

Of the four antiviral A3s, three, A3D, A3F, and A3G, have two cytidine deaminase domains, whereas A3C has one cytidine deaminase domain and inhibits HIV-1 only weakly (17, 24, 25). These observations led us to hypothesize that synthetic double domain variants of the weakly active A3C would be more active than their single domain counterparts. To determine the antiviral effects of single-domain versus double domain A3s, we created synthetic tandem domain A3Cs, called A3C-A3C here, using both A3C_S188,_ the variant that is most common in the human population, and A3C_I188_, the variant that has increased antiviral activity and is present at a low frequency in humans (17).

We modeled the synthetic tandem deaminase protein after the most closely sequence-related double-deaminase *A3* genes, *A3D* and *A3F*. A3C-A3C constructs were created by fusing two A3C sequences connected with a linker sequence (Arg-Asn-Pro) that is naturally found between the N- and C-terminal domains of A3D and A3F, as well as the natural deletion in the N terminus of the second domain of A3C such that the C-terminal domain of A3C-A3C begins at the fifth amino acid of A3C, a methionine (see Fig. S1 in the supplemental material).

10.1128/mBio.00737-20.1 FIG S1 Clustal Omega amino acid alignment of A3C-A3C and the closely related A3 A3F. The “RNP” (arginine-asparagine-proline) amino acid sequence that links the two deaminase domains is highlighted in yellow. The end of the first domain and the beginning of the second domain of A3C-A3C are delineated with a red arrow. The isoleucine human polymorphism in each of the domains is shown in blue text. The conserved A3 cytidine deaminase motif, His-X-Glu-X_23–28_-Cys-Pro-X_2-4_-Cys, is highlighted in gray. The essential glutamic acid that is necessary for deaminase activity is highlighted in green text. Asterisks indicate positions which have a conserved residue between A3C-A3C and A3F; a colon denotes conservation between groups of strongly similar properties; a period indicates conservation between groups of weakly similar properties. Download FIG S1, PDF file, 0.2 MB. Copyright © 2020 McDonnell et al. 2020 McDonnell et al. This content is distributed under the terms of the Creative Commons Attribution 4.0 International license.

**FIG 1:**
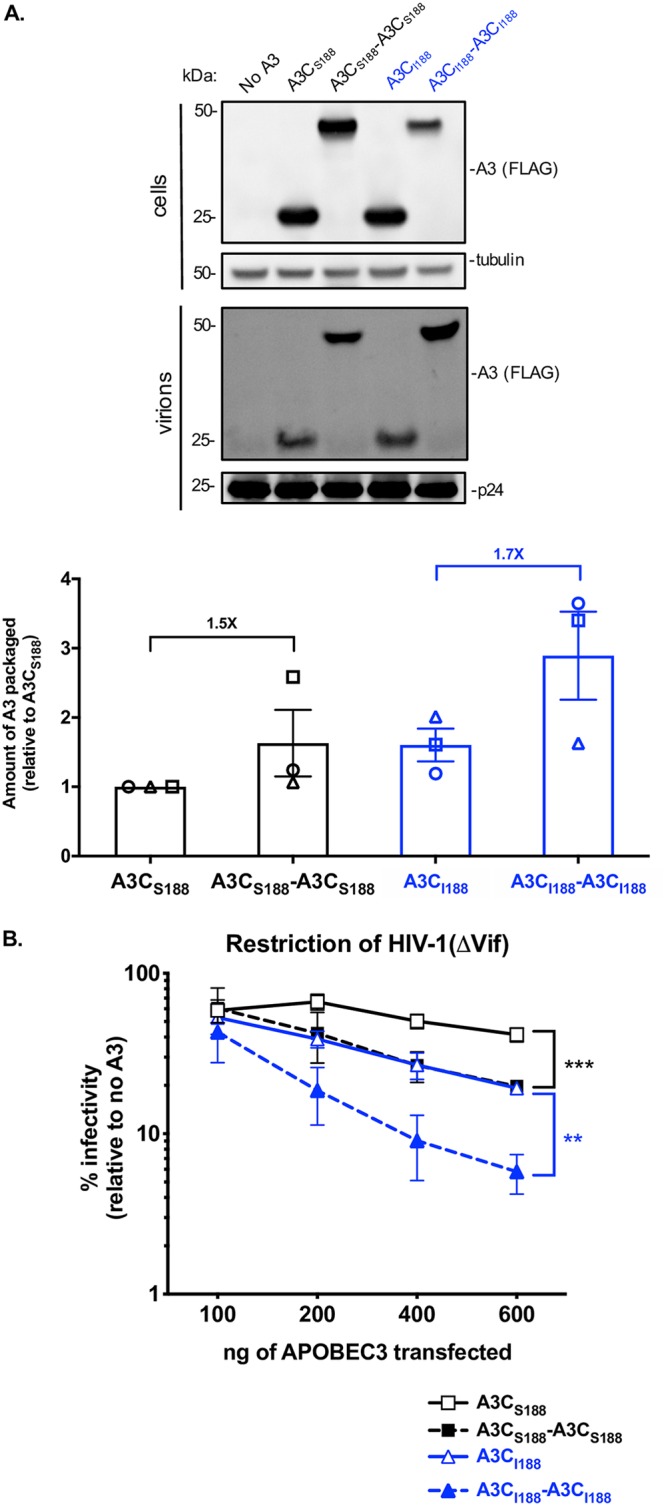
A3C tandem domain proteins have increased antiviral activity and are packaged more extensively than their single domain counterparts. (A) Intracellular expression and virion packaging of A3C variants. HIV-1ΔEnvΔVif provirus was cotransfected into 293T cells with each A3C variant. Each variant is epitope-tagged with a 3X-FLAG tag. (Top) Western blot of cellular lysates probed with anti-FLAG antibody showing intracellular expression levels for A3s and for tubulin as a loading control. (Middle) Western blot of proteins in the pelleted virions probed with anti-FLAG antibody for A3 levels and anti-p24^gag^ for normalization. An empty vector condition (labeled “No A3”) was used as a negative control. The Western blot shown is representative of results from three biological replicates. (Bottom) Bar graph showing quantification of A3 protein packaged into virions from three biological replicates (each represented by a different shape [circle, triangle, or square]). A3 packaged was calculated by dividing the abundance of A3 in the virions normalized to p24^gag^ by the level of A3 expression in the cell normalized to tubulin. The amount of A3 packaged is reported relative to A3C_S188_, whose level was set to a value of 1. The fold increases in packaging of the double domain A3C relative to the indicated examples of the A3C single domain are noted above the bar graph. Error bars represent standard errors of the means (SEM) of results from the three biological replicates (± SEM). (B) Percent infectivity of HIV-1ΔEnvΔVif pseudotyped with VSV-G and the indicated amounts of transfected plasmids of A3C variants is plotted relative to a no-A3 control. A3C_S188_ (black open squares, solid line), A3C_S188_-A3C_S188_ (black closed squares, dotted line), A3C_I188_ (blue open triangles, solid blue line), and A3C_I188_-A3C_I188_ (blue closed triangles, dashed blue line) are compared. Dotted lines denote the double domain A3s, while the solid lines denote single domain A3s. Data points represent means of results from three biological replicates, with each biological replicate consisting of triplicate infections. Error bars show the SEM. Statistical differences were determined by unpaired *t* tests used for comparisons between A3C_S188_ and A3C_S188_-A3C_S188_ (black bracket) and between A3C_I188_ and A3C_I188_-A3C_I188_ (blue bracket): **, *P* ≤ 0.01; ***, *P* ≤ 0.001.

We first examined the ability of each epitope-tagged variant to be expressed in cells and packaged into virions in 293T cells. All A3C variants were expressed at similar levels in cells (Fig. 1A, top), albeit with somewhat lower expression for the A3C_I188_-A3C_I188_ protein. However, the tandem domain A3C proteins were better packaged into virions than the natural single domain A3 proteins. In virions, we found that there was about a 1.6-fold increase in packaging for A3C_S188_-A3C_S188_ compared to A3C_S188_ and about a 2.9-fold increase in packaging of A3C_I188_-A3C_I188_ relative to A3C_S188_ (quantified in the bottom panel in Fig. 1A on the basis of results from three independent experiments).

To evaluate the antiviral activity of these tandem domain proteins, we tested each variant at increasing concentrations in a single-cycle HIV-1ΔVif infectivity assay. As previously shown (17), A3C_S188_ inhibited HIV-1ΔVif only weakly, and A3C_I188_ had increased antiviral activity relative to A3C_S188_ (Fig. 1B; approximately 2-fold greater activity at the highest plasmid dosage). We found that A3C_S188_-A3C_S188_ had approximately 2-fold-increased antiviral activity at every concentration relative to the A3C_S188_ single domain protein (black in Fig. 1B). These levels of enhanced antiviral activity are similar to those seen with the previously characterized A3C_I188_ (17). A3C_I188_-A3C_I188_, however, gained antiviral activity that was 3-fold greater than the level seen with its single domain counterpart A3C_I188_, restricting HIV-1ΔVif to 5.8% infectivity at its highest concentration (blue in Fig. 1B). Thus, these data show that A3C tandem domain proteins have increased antiviral activity relative to their single domain counterparts. This increased antiviral activity of the tandem domain variants relative to the parent single domains (Fig. 1B) largely correlates with the increased packaging of the tandem-domain A3Cs related to the single domain A3Cs (Fig. 1A).

### Mutation of one active site in A3C_S188_-A3C_S188_ increases antiviral activity even further.

**FIG 2:**
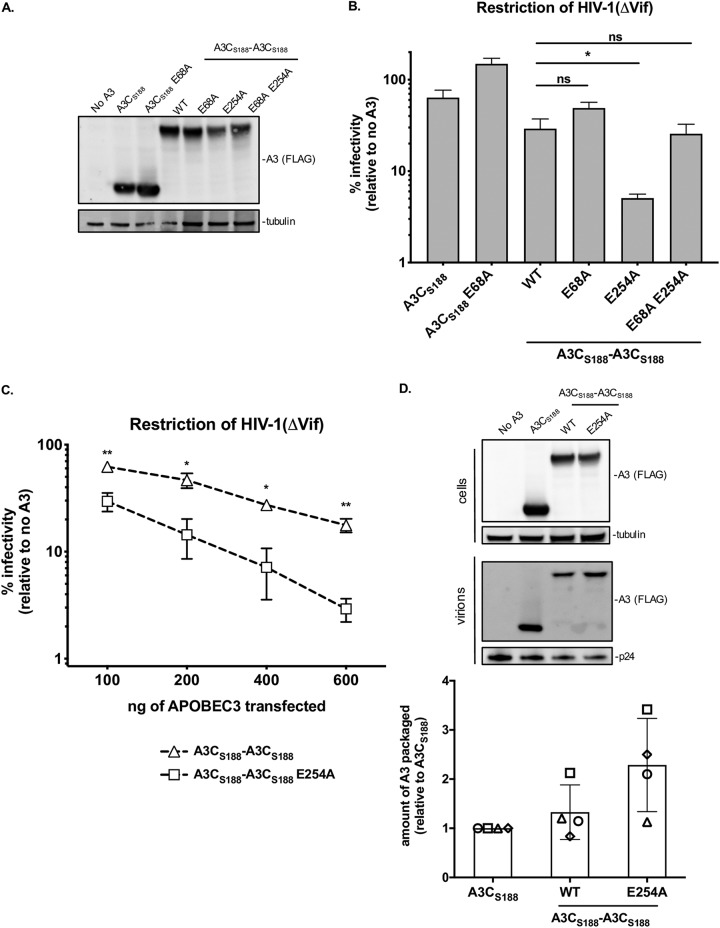
The presence of one functional deaminase domain in a tandem domain A3C protein is optimal for antiviral activity and packaging into virions. (A) Western blotting of intracellular expression levels of active-site point mutation in A3C_S188_-A3C_S188_. “WT” denotes wild-type A3C_S188_-A3C_S188_; “E68A” refers to a mutation in the catalytic deaminase site either in the single domain A3C or at the N terminus of the synthetic tandem domain A3C_S188_-A3C_S188_; “E254A” refers to a mutation in the C terminus of the synthetic tandem domain A3C_S188_-A3C_S188_; “E68A E254A” refers to a double catalytic deaminase site mutant in A3C_S188_-A3C_S188_. Antibodies to FLAG were used to detect A3s, and tubulin was used as a loading control. (B) Single-cycle infectivity assay measuring percent infectivity of each A3C variant and active-site mutant against HIV-1ΔEnvΔVif. Results from each experiment were normalized to a no-A3 control. The bar graph shows means of results from three biological replicates, each with triplicate infections. Error bars represent the SEM. Statistical differences were determined by unpaired *t* tests. *, *P* ≤ 0.05; ns, not significant. (C) Dose response analysis showing the infectivity of HIV-1ΔEnvΔVif in the presence of increasing amounts of transfected plasmids encoding A3C_S188_-A3C_S188_ (open triangles, dotted line) compared to A3C_S188_-A3C_S188_ E254A (open squares, dotted line). Infection was normalized to a no-A3 control. Data points represent means of results from three biological replicates, with triplicate infections. Error bars represent SEM. Statistical differences were determined by unpaired *t* tests. *, *P* ≤ 0.05; **, *P* ≤ 0.01. (D) (Top) Representative Western blot of the packaging of A3C_S188_-A3C_S188_ E254A into virions. HIV-1ΔEnvΔVif provirus was cotransfected into 293T cells with A3C variants. Intracellular levels of A3 proteins were determined using a Western blot probed with anti-FLAG antibody for A3 levels and anti-tubulin as a loading control as indicated. Proteins in the pelleted virions on the indicated Western blot were probed with anti-FLAG antibody for A3 levels and anti-p24^gag^ for normalization. A no-A3 control was used a transfection control. (Bottom) Quantification of A3 protein packaged into virions from four biological replicates. The amount of A3 packaged was calculated by dividing the abundance of A3 in the virions normalized to p24^gag^ by the level of A3 expression in the cell normalized to tubulin. The amount of A3 packaged is reported relative to A3C_S188_, whose level was set to a value of 1. Each plotted shape (circle, square, triangle, or diamond) represents quantification of A3 from one biological replicate, and error bars represent SEM.

A3C_S188_-A3C_S188_ contains two cytidine deaminase motifs within the A3 conserved amino acid sequence His-X-Glu-X_23–28_-Cys-Pro-X_2-4_-Cys (Fig. S1). In A3F and A3G, the C-terminal catalytic domain exerts the key enzymatic activity, while the N-terminal catalytic domain mediates packaging into virions (7, 22, 23). Therefore, we next asked if the increase in antiviral activity of A3C_S188_-A3C_S188_ is due to two active cytidine deaminase sites and whether or not the enzymatic functions of A3C are important for restriction. We created active site point mutations by changing the essential glutamic acid to an alanine in each domain, E68A and E254A, respectively, and found that these changes did not affect protein expression levels in 293T cells (Fig. 2A). As expected, when the glutamic acid was mutated to an alanine in the single domain A3C_S188_ (called A3C_S188_ E68A), the protein completely lost its relatively weak antiviral activity (Fig. 2B). In contrast and unexpectedly, when the C-terminal active site of A3C_S188_-A3C_S188_ was mutated, creating A3C_S188_-A3C_S188_ E254A, the antiviral activity instead increased by 5.8-fold (Fig. 2B). On the other hand, when the N-terminal active site was inactivated in A3C_S188_-A3C_S188_ (called A3C_S188_-A3C_S188_ E68A), the antiviral activity did not change significantly (Fig. 2B), suggesting that this site is not necessary for the antiviral activity but that the active site at position 254 inhibits antiviral activity.

We further tested the abilities of A3C_S188_-A3C_S188_ E254A and A3C_S188_-A3C_S188_ to inhibit HIV-1ΔVif in a dose-response experiment by increasing the amount of *A3* plasmid. At the highest concentrations of plasmid transfected, A3C_S188_-A3C_S188_ E254A was able to reduce the infectivity to 3%, while the wild-type A3C_S188_-A3C_S188_ reduced infectivity only to 18% (Fig. 2C). This potent antiviral activity of the A3C_S188_-A3C_S188_ E254A mutant suggests that having only one functional active site increases its antiviral effect.

In order to test if the increase in antiviral activity of the A3C_S188_-A3C_S188_ E254A mutant is also linked to increased packaging into virions, we examined the amount of packaged A3 via Western blotting. Compared to the intracellular expression levels, A3C_S188_-A3C_S188_ and A3C_S188_-A3C_S188_ E254A were expressed at relatively equal amounts (Fig. 2D). However, evaluation of A3 packaged into virions revealed a 2.3-fold increase in A3C_S188_-A3C_S188_ E254A packaging compared to A3C_S188_ (Fig. 2D). This finding suggests that having only one, rather than two, functional catalytic sites increases the packaging and antiviral activity of A3C_S188_-A3C_S188_.

Both cytidine deaminase-dependent and cytidine deaminase-independent mechanisms of A3 inhibition of HIV-1 were described previously (1, 7–11). The antiviral activity of A3C_S188_ requires an intact deaminase motif, as demonstrated by the total loss of restriction in A3C_S188_ E68A (Fig. 2B). However, when both active sites were mutated to make a catalytically inactive A3C_S188_-A3C_S188_, we still saw 3.8-fold restriction of HIV-1ΔVif—a level of activity indistinguishable from that seen with the wild-type A3C_S188_-A3C_S188_ (Fig. 2B). This suggests that the synthetic tandem domain A3C_S188_-A3C_S188_ functions as an antiviral protein in a cytidine deaminase-independent manner.

We also asked if A3C_I188_-A3C_I188_ would show increased antiviral activity with only one active deaminase domain. Therefore, we mutated each of the essential glutamic acids to an alanine in A3C_I188_-A3C_I188_ and tested for their ability to restrict HIV-1ΔVif. All of these A3C_I188_-A3C_I188_ active-site mutants were expressed to similar levels in 293T cells (Fig. S2A). When the C-terminal active site was mutated, A3C_I188_-A3C_I188_ E254A lost antiviral activity (2.2-fold restriction) compared to the wild-type A3C_I188_-A3C_I188_. Similarly to A3C_S188_-A3C_S188_, the N-terminal active-site mutant had antiviral activity that was not statistically significant from that seen with the wild-type A3C_I188_-A3C_I188_ (Fig. S2B). However, in contrast to A3C_S188_-A3C_S188_, the C-terminally inactive mutant A3C_I188_-A3C_I188_ E254A and the doubly inactive mutant A3C_I188_-A3C_I188_ E68A E254A were indistinguishable in their antiviral activities, suggesting that A3C_I188_-A3C_I188_, like A3F and A3G, uses only one catalytic site. Thus, we speculate that A3C_I188_-A3C_I188_ is already optimized for its most potent restriction.

10.1128/mBio.00737-20.2 FIG S2 (A) Western blot analysis of intracellular expression levels of active-site point mutation in A3C_I188_-A3C_I188_. “WT” denotes wild-type A3C_I188_-A3C_I188_; “E68A” refers to a mutation in the catalytic deaminase site in either the single-domain A3C or N terminus of the synthetic tandem-domain A3C_I188_-A3C_I188_; “E254A” refers to a mutation in the C terminus of the synthetic tandem-domain A3C_I188_-A3C_I188_; “E68A E254A” refers to a double catalytic deaminase site mutant in A3C_I188_-A3C_I188_. Antibodies to FLAG were used to detect A3s, and tubulin was used as a loading control. (B) Single-cycle infectivity assay measuring percent infectivity of each A3C variant and active-site mutant against HIV-1ΔEnvΔVif. Results from each experiment were normalized to those from a no-A3 control. The bar graph shows means results of three biological replicates, each performed with triplicate infections. Error bars represent the SEM. Statistical differences were determined by unpaired *t* tests and are indicated as follows: *, *P* ≤ 0.05; **, *P* ≤ 0.01; ns, not significant. Download FIG S2, PDF file, 0.1 MB. Copyright © 2020 McDonnell et al. 2020 McDonnell et al. This content is distributed under the terms of the Creative Commons Attribution 4.0 International license.

### The A3C synthetic tandem domain antiviral activity does not correlate with hypermutation activity.

**FIG 3:**
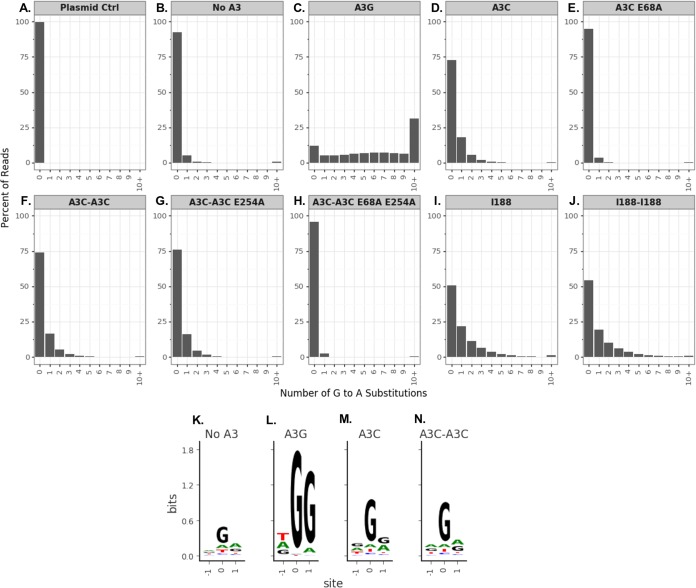
The percentage of G-to-A mutations does not increase in A3C tandem domain proteins. (A to J) SUPT1 cells were infected with HIV-1 virus containing A3 and harvested for viral cDNA 12 h postinfection. Paired-end sequencing reads were analyzed for G-to-A mutations in a region of *pol*. (A and B) A plasmid control (Plasmid Ctrl) in the absence of an infection (A) was used as a sequencing control, and a no-A3 sample (B) was used to distinguish background mutations, including reverse transcriptase mutations. (C to J) Data are shown as bar graphs representing percentages of reads corresponding to the number of G-to-A substitutions in each read for A3 tested. (C) A3G. (D) A3C_S188_. (E) A3C_S188_ E68A. (F) A3C_S188_-A3C_S188_. (G) A3C_S188_-A3C_S188_ E254A. (H) A3C_S188_-A3C_S188_ E68A E254A. (I) A3C_I188_. (J) A3C_I188_-A3C_I188_. G-to-A mutations were calculated as the average frequencies of G-to-A mutations between the technical replicates, and read counts are shown as the sums of the reads for both replicates. (K to N) Extracted sequence information for all mutations (and neighboring bases) in the no-A3 (K), A3G (L), A3C_S188_ (M), or A3C_S188_-A3C_S188_ (N) samples on viral cDNA. The constructed three-nucleotide logo plots are centered on the site of substitution, such that site 0 is fixed as the site of substitution and the −1 and +1 sites indicate the nucleotides immediately 5′ and 3′, respectively, of any substitution in these samples. The letter height shows the enrichment of that nucleotide in the sample compared to the plasmid control, measured in bits representing information content.

In order to directly test the ability of the A3C-A3C variants to hypermutate HIV-1 ssDNA, we developed an assay to assess G-to-A mutations by sequencing large numbers of unintegrated viral DNA products 12 h postinfection. This assay utilizes primers with unique barcodes designed to distinguish between A3 mutations and Illumina sequencing error (26). A plasmid-only control was used to identify sequencing error rates and the frequency of mutations acquired during PCR. A “no-A3” control was used to distinguish mutations made by reverse transcriptase from those introduced by the A3 variants and any residual A3 activity in 293T cells. Each sequencing read spanned the same length of HIV-1 *pol*. The number of unique sequencing reads for each condition ranged from 90,833 to 905,916. Only 0.2% of the reads from the plasmid-only control had any G-to-A mutations (Fig. 3A). A total of 7% of the reads from the infection without any added A3 had a G-to-A mutation (Fig. 3B), presumably the result of mutations from reverse transcriptase or residual A3 activity from 293T cells. In contrast, A3G showed the highest rate of G-to-A hypermutation, with 88% of the reads having at least 1 mutation and with over 30% of the reads having 10 or more mutations. A3G also induced a distribution of templates having 2 to 9 of such mutations (Fig. 3C). In contrast, A3C_S188_ caused fewer G-to-A mutations than A3G, with 27% of that reads containing at least one mutation and 9% having two or more mutations, as shown with the shift toward the left in the bar graphs relative to A3G (comparing Fig. 3C to D). As expected from the single-cycle infectivity assay (Fig. 2B), the mutational burden induced by the A3C_S188_ E68A active-site mutant was not above background levels—only 5% of reads had any G-to-A mutations, and 2% of reads had two or more of such mutations (Fig. 3E).

Consistent with the results indicating that much of the antiviral activity of A3C_S188_-A3C_S188_ did not depend on the presence of an active cytidine deaminase (Fig. 2B), we found that A3C_S188_-A3C_S188_ did not increase the number of G-to-A mutations compared to A3C_S188_ (compare Fig. 3F and D). For the A3C_S188_-A3C_S188_ and A3C_S188_ samples, 26% and 27% of reads had one or more G-to-A mutations, respectively, and the distributions of the G-to-A mutation plots were nearly identical. Interestingly, 24% of the reads from the A3C_S188_-A3C_S188_ E254A sample had a G-to-A mutation, resulting in a distribution similar to that seen in the bar graph for A3C_S188_-A3C_S188_ (Fig. 3G and F, respectively). This finding complements the increase in packaging results seen in Fig. 2D. As an additional control, A3C_S188_-A3C_S188_ E68A E254A had a G-to-A substitution rate below background levels (Fig. 3H).

Previous studies have shown that the increase in antiviral activity of A3C_I188_ is due to an increase in enzymatic activity (17). That finding was replicated here with a shift toward the right in the A3C_I188_ bar graph compared to A3C_S188_ (comparing Fig. 3I and D) such that 49% of reads had at least one G-to-A mutation. A3C_I188_-A3C_I188_, despite having more antiviral activity than A3C_I188_ (Fig. 1A), did not have a higher percentage of mutations—49% of reads had at least one mutation in A3C_I188_ and 45% at least one in A3C_I188_-A3C_I188_ (Fig. 3I and J). In sum, the hypermutation activities for the double-domain A3Cs appear to have had distributions nearly identical to those seen with the corresponding single domain A3Cs. Taken together, these deep-sequencing data of A3 hypermutation support our earlier conclusion from the double active-site mutations (Fig. 2B) that the increase in antiviral activity was not due to an increase in enzymatic activity.

In addition, we determined if the nucleotide preferences for G-to-A mutations were changed by addition of a second cytidine deaminase domain to A3C. As expected (27), A3G has a strong preference of 5′-GG-3′ on the positive-sense strand, with the −1 site having equal preferences among the nucleotides present (Fig. 3K). However, the degrees to which A3C_S188_ and A3C_S188_-A3C_S188_ showed a lack of preference for the −1 and +1 positions were similar (Fig. 3M and N), further supporting the idea that an increase in mutation frequency does not explain the different antiviral activities of A3C_S188_ and A3C_S188_-A3C_S188_.

### A3C_S188_-A3C_S188_ tandem domain variants reduce the accumulation of reverse transcription products.

**FIG 4:**
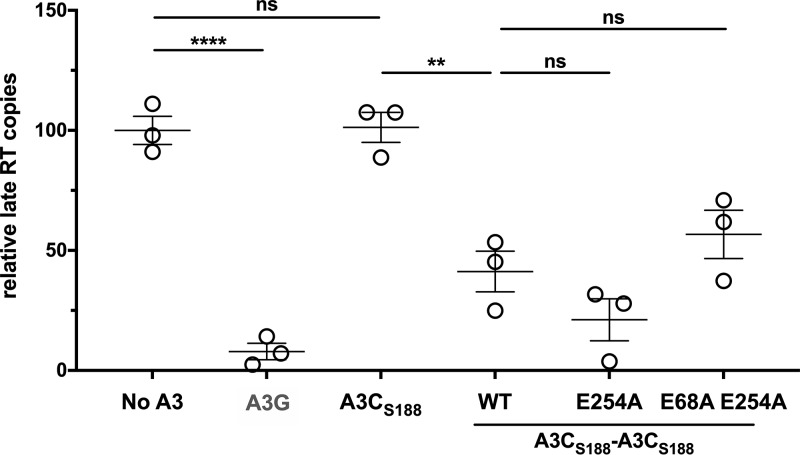
A3C tandem domain proteins operate in a deaminase-independent mechanism to inhibit reverse transcription products. Data represent numbers of copies of late reverse transcription products after infection relative to an infection of virus produced with no A3 (set to 100). SUPT1 cells were infected with HIV-1ΔEnvΔVif and either no A3 or A3 to test for inhibition of HIV-1 reverse transcription. At 18 h later, viral cDNA was harvested and the levels of HIV-1 late reverse transcription products were assayed by qPCR. The experiment was done with three separate biological replicates, with each shape (circle, triangle, or square) representing a normalized mean of results from qPCR technical duplicates for the respective biological replicate. Each sample has been adjusted for equal levels of viral infection and a nevirapine control to subtract DNA carryover. Error bars indicate the SEM of results from three biological replicates. Statistical differences were determined by unpaired *t* tests. **, *P* ≤ 0.01; ****, *P* ≤ 0.0001; ns, not significant.

Since we did not see an increase in G-to-A mutations in the A3C-A3C variants relative to A3C alone, we suspected that, similarly to results reported previously from studies performed with an A3G active-site mutant (9–11), the A3C-A3C tandem domain proteins might decrease levels of reverse transcriptase products independently of hypermutation. To test this hypothesis, we used quantitative PCR (qPCR) to quantify late reverse transcription (RT) products from unintegrated viral DNA. Virus produced in the presence of A3G showed a significant decrease in relative levels of late RT products compared to the no-A3 control, while A3C_S188_ had levels of late RT products equivalent to those seen with the no-A3 control (Fig. 4). However, the amount of RT products produced from virus made in the presence of A3C_S188_-A3C_S188_ was significantly (2.5-fold) reduced relative to that made in the presence of A3C_S188_ (Fig. 4). A3C_S188_-A3C_S188_ E254A showed levels of late RT products equivalent to those seen with A3C_S188_-A3C_S188_, further confirming that the increase in antiviral activity came from the increase in packaging into virions (Fig. 4; see also Fig. 2C and D). Finally, there was no significant difference in the levels of late RT products of A3C_S188_-A3C_S188_ and A3C_S188_-A3C_S188_ E68A E254A, suggesting that inhibition of RT was likely the mechanism by which these variants acted (Fig. 4; see also Fig. 2B). These findings further support the hypothesis that A3C super restriction factors function through a distinct mechanism to restrict HIV-1 compared to A3C single domain proteins.

### Tandem domain variants of A3C form larger higher-order complexes in cells than their native single domains.

**FIG 5:**
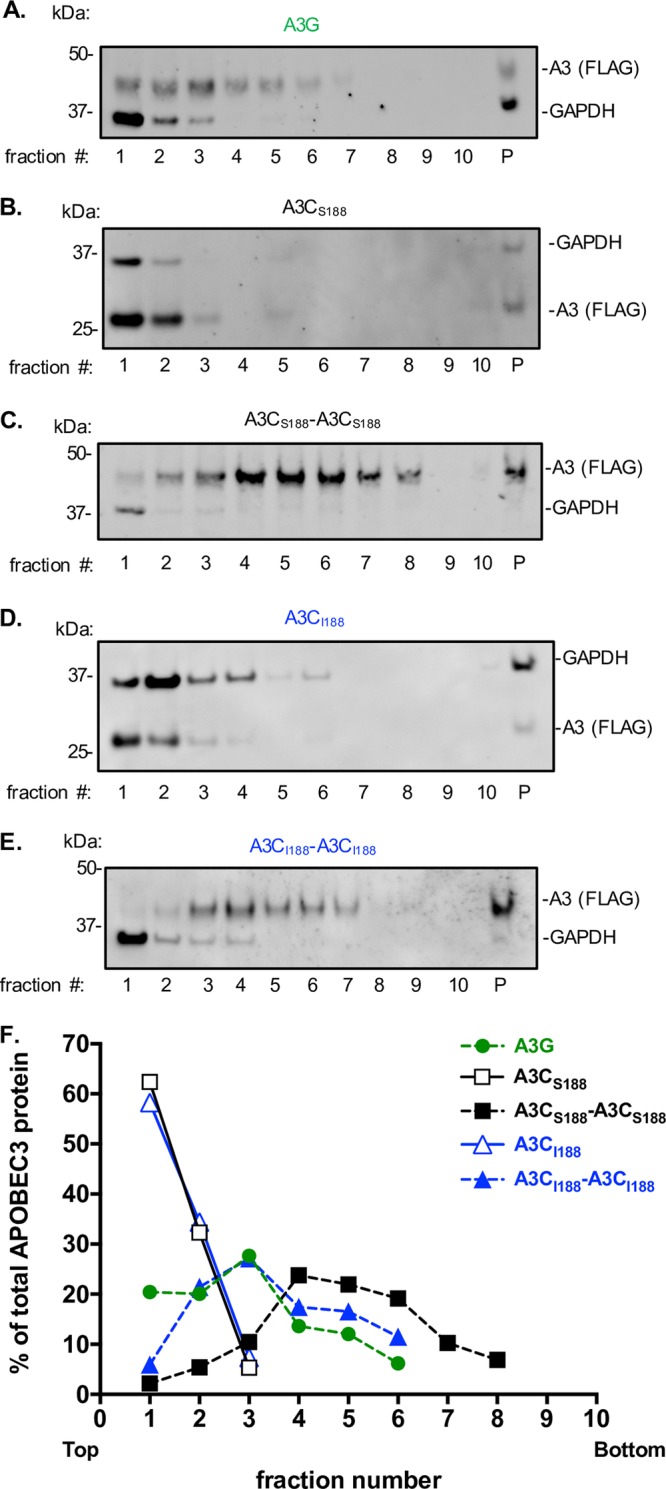
A3C double-deaminase-domain proteins form larger complexes than the single domains. (A to E) Velocity sedimentation and sucrose fractionation of A3C variants. Cell lysates from 293T cells transfected with A3 were subjected to velocity sedimentation fractionation (lanes 1 to 10; lane P = pellet), and 2.5% of each fraction was analyzed by Western blotting. (A) A3G. (B) A3C_S188_. (C) A3C_S188-S188_. (D) A3C_I188_. (E) A3C_I188-_A3C_I188_. Each Western blot was probed with anti-FLAG to analyze A3 levels and with anti-GAPDH to mark the soluble fraction. Higher fraction numbers indicate positions that are lower on the gradient and represent increasingly larger complexes. (F) Quantification of the percentage of the total A3 protein in each fraction. The relative abundance of A3 in each fraction from the sucrose gradient Western blots was calculated as a percentage of the total APOBEC3 protein found in all the fractions combined. Dotted lines denote the double domain A3s, while the solid lines denote single domain A3s. A3G is shown in green circles, A3C_S188_ in open black squares, A3C_S188_-A3C_S188_ in closed black squares, A3C_I188_ in open blue triangles, and A3C_I188_-A3C_I188_ in closed blue triangles.

The ability of A3G to oligomerize in cells has been correlated with its antiviral activity because this oligomerization leads to increased packaging into virions (28, 29). The A3G oligomerization state also affects its binding to ssDNA and its catalytic activity (30). More specifically, A3G present in lower-molecular-mass complexes has the ability to deaminate ssDNA (30, 31), while A3G residing in high-molecular-mass complexes is inactive, hinders rapid deamination of ssDNA, and is hypothesized to instead form a roadblock to inhibit reverse transcription (30, 31). Because we observed that the A3C-A3C variants inhibited late RT products (Fig. 4) rather than inducing hypermutation (Fig. 3), we examined the ability of each of the A3C variants to form high-molecular-weight complexes in a velocity sedimentation experiment. As expected from previous reports (28, 32), A3G formed both lower-order and higher-order complexes as demonstrated by its presence in the top and middle fractions of a sucrose gradient (Fig. 5A). In contrast, both A3C_S188_ and A3C_I188_ were found in the top fractions of the gradient, overlapping with the GAPDH (glyceraldehyde-3-phosphate dehydrogenase) soluble control (Fig. 5B and D, respectively). This suggests the A3C single domain protein does not form higher-order complexes, unlike A3G. In contrast, we found that both A3C_S188_-A3C_S188_ and A3C_I188_-A3C_I188_ formed complexes that migrated farther down the sucrose gradient, similarly to A3G (Fig. 5C and E, respectively). However, A3C_S188_-A3C_S188_ migrated even farther down the sucrose gradient than A3G. Furthermore, both A3C_S188_-A3C_S188_ and A3C_I188_-A3C_I188_ had less protein in the top fractions than both A3G and their single domain counterparts (Fig. 5F). Together, these findings suggest that the A3C tandem domain variants reside primarily in larger higher-order complexes.

### A3C tandem domain proteins are largely resistant to viral antagonism by Vif.

**FIG 6:**
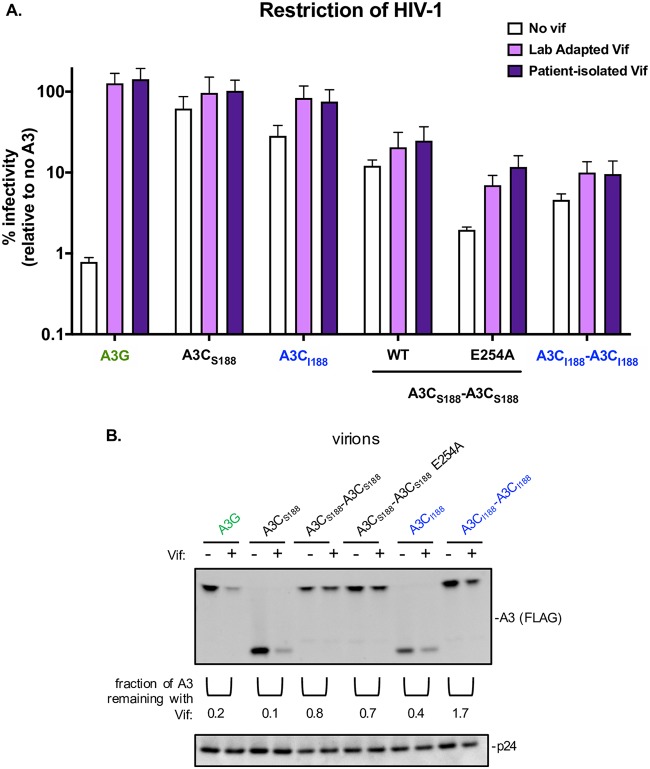
A3C tandem domain variants are resistant to viral antagonism. (A) Single-cycle infectivity assay performed in the presence of HIV-1ΔEnvΔVif (white), HIV-1ΔEnv +LAI Vif (Lab Adapted Vif; light purple), and HIV-1ΔEnv plus 1203 Vif (Patient-isolated Vif; dark purple). Results from each experiment were normalized to a no-A3 control. The bar graph shows means of results from three biological replicates, each with triplicated infections. Error bars represent SEM. (B) Western blot of the packaging of A3C variants into virions. Either HIV-1ΔEnvΔVif or HIV-1ΔEnv plus LAI Vif provirus was cotransfected into 293T cells with A3C variants. Proteins in the pelleted virions are shown in a Western blot probed with anti-FLAG antibody for A3 levels and anti-p24^gag^ as a loading control. Densitometry calculations were performed to determine the fraction of A3 remaining in the presence of Vif shown under the anti-FLAG Western blot with pairs without (−) and with (+) Vif indicated for each A3. The Western blot shown is representative of three biological replicates.

The most effective super restriction factors would need to both increase in antiviral activity and overcome viral antagonism. Therefore, we next examined the ability of the A3C-A3C super restriction factors to escape viral antagonism. To test if HIV-1 Vif from two different strains could degrade novel A3C tandem domain protein targets, we performed a single-cycle infectivity assay using an HIV provirus containing a *Vif* gene from either a lab-adapted strain (LAI) or a primary-isolate *Vif* (patient identifier [ID] 1203) that has been previously shown to degrade A3H and A3C variants (33). In the absence of the viral antagonist Vif, A3G potently inhibited HIV-1ΔVif (Fig. 6A), and, as expected, the presence of either HIV-1 LAI Vif (light purple bars) or HIV-1 ID1203 Vif (dark purple bars) led to full antagonism of A3G (Fig. 6A). Whereas A3C_S188_ and A3C_I188_ inhibited HIV-1ΔVif, the presence of Vif fully antagonized their antiviral activity (Fig. 6A). In contrast, each of the A3C tandem variants was partially resistant to Vif degradation as infectivity was not completely restored (Fig. 6A). Even in the presence of Vif, A3C_S188_-A3C_S188_ still restricted HIV-1 with an LAI Vif to 20% infectivity and ID1203 Vif to 24%. Furthermore, A3C_S188_-A3C_S188_ E254A restricted HIV-1 to 7% and 12% infectivity (LAI Vif and primary-isolate Vif, respectively), and A3C_I188_-A3C_I188_ inhibited infection to 10% with both HIV-1 Vifs (Fig. 6A).

As expected from the inability of HIV-1 Vif to antagonize the A3C-A3C tandem-domain proteins (Fig. 6A), we found that HIV-1 Vif did not decrease the amount of A3C-A3C packaged into virions (Fig. 6B). We used Western blotting to evaluate levels of A3 proteins packaged into virions in the presence and absence of Vif (Fig. 6B). As a control, we used A3G, A3C_S188_, and A3C_I188_ as naturally found A3s that are degraded by HIV-1 Vif. Using densitometry, we quantified and compared the fractions of A3 probed in the presence of an HIV-1 provirus containing a deletion in the *Vif* gene to a LAI HIV-1 provirus retaining the *Vif* gene. As expected, A3G had a 0.2 fraction of A3 remaining in the presence of Vif, A3C_S188_ had 0.1, and A3C_I188_ had 0.4, since HIV-1 Vif is able to degrade the naturally found A3s (Fig. 6B). Furthermore, these data support the rescue of infection observed in the single-cycle infectivity assay (Fig. 6A). We found that A3C_S188_-A3C_S188_, A3C_S188_-A3C_S188_ E254A, and A3C_I188_-A3C_I188_ were packaged into budding virions in the presence of HIV-1 Vif, with the fractions of A3 remaining in the presence of Vif seen to be 0.8, 0.7, and 1.7, respectively (Fig. 6B). This fraction of A3 remaining is much higher than that seen with the single domain counterparts and supports the data corresponding to the inhibition of HIV-1 infection in the presence of Vif seen in the single-cycle infectivity assay (Fig. 6). Since HIV-1 Vif from two divergent strains was unable to fully antagonize these A3C-A3C variants, our data show that a synthetic tandem domain version of an A3 protein can both increase its potency and allow it to resist antagonism by HIV-1 Vif.

## DISCUSSION

Although human A3C can weakly inhibit HIV-1 replication, here we show that a super restriction factor can be created by linking two A3C sequences. These tandem domain proteins both increase anti-HIV-1 activity and yield a restriction factor that is partially resistant to antagonism by HIV-1 Vif. We have shown that this increase in antiviral activity is mostly explained by increased packaging of tandem domain A3s into budding virions. Moreover, we found that A3C_S188_-A3C_S188_ appears to use both a cytidine deaminase-dependent mechanism and a cytidine deaminase-independent mechanism for HIV-1 restriction. However, we were able to further increase the antiviral activity of A3C_S188_-A3C_S188_ by mutating the C-terminal active site (A3C_S188_-A3C_S188_ E254A). We found that the A3C-A3C tandem domain proteins did not show an increase in mutation frequency compared to their single domain counterparts but rather that the dominant mechanism of restriction operated through inhibition of reverse transcription. Additionally, all the A3C tandem domain variants were found to be resistant to HIV-1 Vif degradation. Together, these data point toward a selective advantage of double domain versus single domain A3 proteins against a viral target and support the idea that additional potency and escape from viral antagonism can be derived from combinations of APOBEC3 domains that have not been sampled in nature.

### Two-domain A3C proteins are packaged at higher levels than single domain A3C proteins.

In order for the A3 proteins to be antiviral, they must be packaged into virions to function during the reverse transcription process that occurs in the target cell. We found that the increases in the antiviral activity of the A3C tandem domain proteins relative to their single domain counterparts closely paralleled the increased activity of A3 packaged into budding virions (Fig. 1 and 2). However, the variability of the packaging assay prevents us from concluding that packaging alone is responsible for the increased antiviral activity. Nonetheless, each of the A3C-A3C variants formed larger higher-order complexes than the corresponding single domain proteins (Fig. 5) and it was shown previously that oligomerization is important for packaging of A3G into virions (29). A3H is an interesting exception to the idea that double-cytidine-deaminase A3 proteins have better oligomerization characteristics than single-cytidine-deaminase A3 proteins because it is the most distantly related A3, with only one deaminase domain that never duplicated or recombined to form a double-deaminase protein (5). However, similarly to A3G and the A3C-A3C variants, A3H can form large complexes in sucrose gradients, and yet these complexes are RNA dependent (34, 35). Also, A3H, like A3C, is polymorphic in human populations; however, A3H haplotype II confers strong antiviral activity (25). Recent structural work has shown that A3H binds to RNA to make functional dimers (35–37). This suggests that A3H perhaps evolved an independent mechanism to form higher-order structures that is dependent on RNA but that A3C is unable to do this unless the second cytidine deaminase domain is artificially engineered. Furthermore, we can speculate that nature has selected for single domain and double domain A3s because of the selective advantages of both monomer and dimer populations for deaminase-dependent activity as well as a population of larger-order complexes for deaminase-independent activity.

### Deaminase-dependent and deaminase-independent mechanisms of super restriction factor antiviral activity.

Naturally found A3 proteins primarily restrict HIV-1 through hypermutation of ssDNA intermediates during reverse transcription. Studies have reported that A3G and A3F can also function through a cytidine deaminase-independent mechanism to restrict HIV-1 (7–10). However, hypermutation rather than steric inhibition is the primary mode of restriction (7, 8, 11). Furthermore, A3G forms both low-order and high-order complexes in cells, but the A3G residing in the higher-order complexes has been shown to have hindered deaminase activity (30). While A3G residing in the smaller complexes appears to be deaminating ssDNA targets, these large A3G complexes have been hypothesized to form a roadblock to inhibit reverse transcription (30). Interestingly, the A3C_S188_-A3C_S188_ double-inactive-site mutant (A3C_S188_-A3C_S188_ E68A E254A) still retained antiviral activity that, in fact, was indistinguishable from the activity seen with wild-type A3C_S188_-A3C_S188_ (Fig. 2B). The A3-mediated hypermutation assay data added to these findings by showing that the tandem domain A3 proteins did not have more enzymatic activity than their single domain counterparts (Fig. 3). Rather, both A3C_S188_-A3C_S188_ and A3C_S188_-A3C_S188_ E254A inhibited the total late RT products more extensively than their A3C single domain counterparts (Fig. 4). Each of these results is also consistent with a lack of total A3 in the top fraction of the A3C tandem domains (Fig. 5). This finding supports the hypothesis that these large higher-order complex contribute to hindering reverse transcription in a deaminase-independent mechanism. Thus, we found that these A3C-A3C variants gained a new mechanism to restrict reverse transcription: the ability to inhibit HIV-1 reverse transcription, most likely through forming large complexes of oligomers.

### Only one cytidine deaminase domain is active in tandem domain APOBEC3 proteins.

A3G and A3F have two deaminase domains, and yet these naturally found A3s primarily rely on one domain, the C-terminal domain, for their catalytic activity (22, 23). Our finding that the presence of only one active domain resulted in higher levels of antiviral activity than were seen with two active domains (Fig. 2) reflects how the A3F and A3G domains have evolved. However, A3G and A3F do not have enhanced antiviral activity when active-site mutations are created (7, 23). Additionally, the preferred enzymatic domain for A3C_S188_-A3C_S188_ is the N-terminal domain, unlike what was seen with A3F and A3G. Nevertheless, the C-terminal active-site mutant of A3C_S188_-A3C_S188_ can parallel the evolutionary process that the naturally found A3s have undergone. Since A3C_S188_-A3C_S188_ is less potent of a restriction factor than A3C_S188_-A3C_S188_ E254A, this suggests that there is a disadvantage to having two fully active deaminase sites. We speculate that the specialization of the A3 domains could be important for optimal efficiency as an enzyme, such that one domain is primarily used for packaging into virions and oligomerization while the other is important for scanning ssDNA for its deamination activity. Since A3F and A3G have evolved to use only one domain for hypermutation, mutating one catalytic site does not increase antiviral activity. However, A3C_S188_-A3C_S188_ can be further improved upon by mutating the protein such that only one active site is used, A3C_S188_-A3C_S188_ E254A, to recapitulate natural selection seen in A3F and A3G.

### A3C-A3C super restriction factors are mostly resistant to Vif antagonism.

Previous studies have shown that HIV-1 Vif binds and degrades A3C_S188_ and A3C_I188_ (17, 38). One viral protein, Vif, must counteract the antiviral activity of multiple A3s to achieve maximal infectivity for the virus (2). HIV-1 Vif has evolved three separate interfaces in order to degrade A3s: one for binding A3G, another for binding A3H, and a third interface that is able to interact with A3C/A3D/A3F (2). In contrast to the human A3 proteins, HIV-1 has never evolved to antagonize an A3C-A3C tandem domain protein. The synthetic nature of these tandem domain A3s may explain why HIV-1 Vif was not able to completely antagonize any A3C-A3C variants (Fig. 6). Since the two different strains of HIV-1 Vif are approximately 85% identical and cannot fully antagonize A3C-A3C tandem domain proteins, the results suggest the presence of a potential mechanism that the Vif interface previously used to bind to the A3C and that is now occluded by these tandem domain proteins. However, another possibility is that A3C-A3C is packaged so efficiently that Vif is unable to target all the active A3 prior to packaging into virions, although detection of only an ∼1.5-fold increase in packaging of the super restriction factors makes this less likely. In either case, the increased activity and resistance to Vif antagonism of these super restriction factors provide useful insights about the initial constraints of Vif recognition with respect to novel A3 variants.

## MATERIALS AND METHODS

### APOBEC3C tandem-deaminase-domain sequences.

The A3C-A3C synthetic tandem domain was designed on the basis of the most closely related A3, A3F. The N-terminal subunit consists of amino acid residues 1 to 187 of A3C (residues 188 to 190 were deleted) and was codon optimized to distinguish the N terminus from the C terminus. The codon-optimized N terminus was linked to the single domain human A3C at the C terminus using a short amino acid sequence that is found in A3F and A3D, Arg-Asn-Pro (RNP), and that is naturally found in the A3C single domain. The C-terminal domain begins at amino acid 6 (15-nucleotide deletion) to include this RNP sequence and extends to include the remainder of A3C followed by a flexible linker and a triple FLAG (3XFLAG) tag. The A3C-A3C tandem domain was constructed using overlapping extension PCR as described previously (17) and was cloned into a pcDNA4/TO vector (Thermo Fisher; catalog no. V102020) using BamHI/XbaI restriction sites. All point mutations were made using a QuikChange II site-directed mutagenesis kit (Agilent; catalog no. 200524).

### Cell culture and transfections.

HEK293T cells (ATCC, CRL-3216) were cultured in Dulbecco’s modified Eagle’s medium (DMEM) (Gibco; catalog no. 11965092) with 10% HyClone fetal bovine serum (GE Healthcare; catalog no. SH30910.03) and 1% penicillin-streptomycin (Gibco; catalog no. 15140122) at 37°C in a humidified CO_2_ incubator. SUPT1 cells, acquired from ATCC (CRL-1942), were maintained similarly but in RPMI medium (Gibco; catalog no. 11875093), 10% HyClone fetal bovine serum (GE Healthcare; catalog no. SH30910.03), 1% penicillin-streptomycin (Gibco; catalog no. 15140122), and 10 mM HEPES. HEK293T cells were plated in 6-well dishes for transfections at a density of 1.5 × 10^5^ cells/ml.

### Intracellular protein expression and packaging experiments.

Intracellular expression of APOBEC3 proteins during virion production was determined by lysis of the virion-producing 293T cells with NP-40 buffer with protease inhibitor (200 mM NaCl, 50 mM Tris [pH 7.4], 0.5% NP-40 Alternative, 1 mM dithiothreitol [DTT], and Roche Complete Mini, EDTA-free tablets; catalog no. 11836170001). Lysate samples were resolved on a 4% to 12% SDS-PAGE gel using morpholineethanesulfonic acid (MES) buffer and transferred to a nitrocellulose membrane for Western blotting. Anti-FLAG (Sigma; catalog no. F3164), anti-tubulin (Sigma; catalog no. T6199), and anti-p24gag (NIH-ARP; catalog no. 3537) (39, 40) antibodies were used for Western blotting at a dilution of 1:5,000. StarBright Blue 700 goat anti-mouse IgG (Bio-Rad; catalog no. 12005866) was used to detect primary antibodies at a dilution of 1:5,000. The chemiluminescent signals from all Western blots were imaged using a ChemiDocMP imaging system (Bio-Rad), and images were processed using Fiji/ImageJ software to quantify the densitometry for each detected antibody band. Transfections were performed with TransIT-LT1 transfection reagent (Mirus; catalog no. MIR2304) at a reagent/plasmid DNA ratio of 3:1. The following reagent was obtained from Bruce Chesebro and Kathy Wehrly through the NIH AIDS Reagent Program, Division of AIDS, NIAID, NIH: anti-HIV-1 p24 monoclonal (183-H12-5C) (catalog no. 3537).

The amount of A3 packaged into virions was evaluated by cotransfecting 600 ng pcDNA4/TO.A3.3XFLAG and 1,000 ng HIV-1ΔVifΔEnvLuc2 (unless otherwise denoted) in a 6-well plate. At 3 days posttransfection, cell lysates were harvested as described above and 1.5 ml of the supernatant was collected, filtered through a 0.2-μm-pore-size filter, and spun down using a tabletop microcentrifuge for 1 h at maximum speed at 4°C to pellet the virions. The supernatant was aspirated off, and 25 μl of NuPAGE 4× loading dye (Invitrogen; catalog no. NP0007) was added to each sample. Samples were boiled for 10 min at 95°C and loaded on an SDS-PAGE gel.

### Single-cycle infectivity assays.

Single-cycle infectivity assays using HIV-1 were previously described (33). 293T cells were seeded at a density of 1.5 × 10^5^ cells/ml in a 6-well plate. The following day, cells were transfected with 600 ng provirus (HIV-1ΔVifΔEnvLuc2, unless otherwise noted), 100 ng l-VSV-G (vesicular stomatitis virus G), and 400 ng pcDNA4/TO.A3.3XFLAG or pcDNA4/TOPO empty vector unless otherwise indicated. At 72 h later, virus was harvested and normalized for virion production using an RT-qPCR assay, as described previously (41, 42). A volume of virus equivalent to 2,000 mU/ml of reverse transcriptase was used for infection of SUPT1 cells. For infectivity assays, SUPT1 cells were seeded at 2 × 10^4^ cells per well in a 96-well plate in media containing 20 μg/ml DEAE-dextran. At 72 h later, infected cells were lysed in luciferase lysis reagent (Bright-Glo; Promega catalog no. E2610) and luciferase expression was measured on a luminometer (LUMIstar Omega; BMG Labtech). Infectivity of each virus was normalized to 100% based on a no-A3 control. All HIV-1 constructs are based on the LAI strain. A clade B patient-derived Vif (PID: 1203) that is able to antagonize A3H haplotype II was previously described (33).

### Deep sequencing of A3-mediated mutations.

To analyze A3-mediated mutations, 293T cells were seeded at a density of 1.5 × 10^5^ cells/ml in a 6-well plate. The following day, cells were transfected with 600 ng provirus (HIV-1ΔVifΔEnvLuc2), 100 ng l-VSV-G, and 400 ng pcDNA4/TO.A3.3XFLAG or pcDNA4/TO empty vector. Three days later, virions were harvested and quantified similarly to the single-cycle infectivity assay mentioned above. A total of 2 × 10^6^ SUPT1 cells were infected with 500 μl of each Benzonase-treated virus with and without a 10 mM nevirapine control and subjected to spinoculation at 1,100 × *g* for 30 min at 30°C. Twelve hours later, unintegrated viral cDNA was isolated using a Qiagen miniprep kit (QIAprep Spin miniprep kit; catalog no. 27106).

To determine A3-mediated mutations, we used a barcoded Illumina deep-sequencing approach as previously described (26) (explained in more detail at https://jbloomlab.github.io/dms_tools2/bcsubamp.html). This approach attaches unique molecular identifiers (8*N) to each DNA molecule, which enables increased correction of PCR and sequencing errors by determining molecule-specific consensus sequences. A region of *pol* (43, 44) was amplified using KOD Hot Start master mix (Millipore; catalog no. 71842) in a first round of PCR with forward primer CTTTCCCTACACGACGCTCTTCCGATCTNNNNNNNNGACAAGGAACTGTATCCTTTAACTT and reverse primer GGAGTTCAGACGTGTGCTCTTCCGATCTNNNNNNNNCTGGTACAGTTTCAATAGGACTAAT. The first-round PCR was carried out using the following parameters: 95°C for 2 min and 20 cycles of 95°C for 20 s, 70°C for 1 s, 50°C for 10 s, and 70°C for 2 min, followed by 95°C for 1 min and a hold at 4°C. PCR products were cleaned up using AMPure beads (Beckman Coulter; catalog no. A63880) and quantified with a Qubit double-stranded DNA (dsDNA) HS assay kit (Thermo Fisher; catalog no. Q32854). Next, the first-round PCR products were bottlenecked such that each of the uniquely barcoded ssDNA molecules would be read ∼2.9 times during sequencing. A second round of PCR was performed with KOD Hot Start master mix (Millipore; catalog no. 71842) supplemented with 1 mM MgCl_2_ with the following parameters: 95°C for 2 min and 23 cycles of 95°C for 20 s, 70°C for 1 s, 60°C for 10 s, and 70°C for 10 s, followed by a hold at 4°C. Samples were again cleaned up, quantified, pooled, purified via gel electrophoresis, and sequenced on an Illumina MiSeq sequencer, using 2 × 250 paired-end reads. Deep sequencing was carried out in technical replicate under each experimental condition, starting with the initial PCRs.

We used the software package dms_tools2 to align sequencing reads and build consensus sequences for each uniquely tagged DNA molecule (45). Error-corrected reads were compared to the target sequence to determine the number, identity, and surrounding nucleotides of all substitutions in each read. Reads with high numbers of substitutions (>10% of non-G nucleotides) at the junction of the two paired-end reads were removed from the analysis as these substitutions were most often found to be alignment artifacts. Since A3s are known to cause G-to-A substitutions, we initially subsampled our data to look only at G-to-A substitutions. We plotted the frequency of reads in each sample with a given number of G-to-A mutations (0, 1, 2, etc., up to 9 and then 10+) (Fig. 4A to J). Frequencies were calculated as the average frequencies of technical replicates, and read counts are shown as the sum of reads for both replicates.

To investigate if substitutions occurred more frequently in certain nucleotide contexts, we computationally extracted all three-nucleotide sequences centered on a substitution from each sample. This gave us a count for each sample corresponding to how many times each nucleotide was present immediately upstream (site −1) of a substitution, underwent a substitution (site 0), or was present immediately downstream of a substitution (site +1). We then calculated the frequency for each nucleotide at each of these three sites in each sample. Data for technical replicates were combined by averaging the frequencies of each nucleotide at each site across the two replicates for each sample. Next, we calculated background-corrected frequencies for each nucleotide at each site using the frequencies in the plasmid control sample as our background. We based this frequency correction on the “type 2” logos from Gorodkin et al. (46).

If *q_ik_* is the frequency of base k at site i in the sample and *p_ik_* is that frequency in the background, then the background corrected frequency is as follows:

$$c_{\mathrm{ik}}=\frac{q_{\mathrm{ik}}/p_{\mathrm{ik}}}{\sum_{i}q_{\mathrm{ik}}/p_{\mathrm{ik}}}$$

We then calculated the information content of each site as follows:

$$I_{i}=\log_{2}N-\left(-\underset{k\in A}{\sum }c_{\mathrm{ik}}\log_{2}c_{\mathrm{ik}}\right)$$

Where *A* is the set of nucleotides (A, C, G, T) and *N* is the number of elements in *A* (46, 47).

Finally, we calculated the letter height at each site as follows:

$$d_{\mathrm{ik}}=c_{\mathrm{ik}}I_{i}$$

### qPCR assay for HIV late reverse transcription products.

For qPCR analysis of HIV late RT products, 2 × 10^6^ SUPT1 cells were infected with 500 μl of each Benzonase-treated virus with and without a 10 mM nevirapine control and subjected to spinoculation at 1,100 × *g* for 1 h at 30°C. At 18 h postinfection, unintegrated viral cDNA was isolated using a Qiagen miniprep kit (QIAprep Spin miniprep kit; catalog no. 27106). HIV cDNA was amplified with TaqMan gene expression master mix (Applied Biosystems; catalog no. 4369016), J1 FWD (late RT F)—ACAAGCTAGTACCAGTTGAGCCAGATAAG, J2 REV (late RT R) GCCGTGCGCGCTTCAGCAAGC, and LRT-P (late RT probe)—6-carboxyfluorescein (FAM)-CAGTGGCGCCCGAACAGGGA-6-carboxytetramethylrhodamine (TAMRA) (48, 49). Data were acquired on an ABI QuantStudio5 real-time (qPCR) machine and analyzed on Prism software.

### Velocity sedimentation.

For analysis of A3 migration on a sucrose gradient, 293T cells were harvested 72 h posttransfection (1,000 ng of A3 per well in a 6-well plate) using a low-salt-and-low-EDTA buffer (0.2 M HEPES [pH 7.9], 0.1 M NaCl, 0.01 MgCl_2_, 0.002 EDTA [pH 8.0], 3.5% Triton X-100) (50) with protease inhibitor cocktail (Roche; catalog no. 11836170001). For each sample, 50 μl of lysate was layered on a step gradient (10%, 15%, 40%, 50%, 60%, 70%, and 80% sucrose in lysis buffer) and subjected to velocity sedimentation in a Beckman MLS50 rotor at 45,000 rpm (163,000 × *g*) for 37 min at 4°C, as described previously (34). Gradients were fractioned (400 μl) from top to bottom and analyzed via Western blotting.

### Data accessibility.

The sequencing reads were uploaded to the NCBI SRA with BioProject accession number PRJNA605864 and run accession numbers SRR11059567 to SRR11059589. The computational pipeline used to analyze the sequencing data and generate Fig. 4 is available on GitHub (https://github.com/jbloomlab/SuperRestrictionFactor_Hypermutation).
