# Supplementary material for: APOBEC3C Tandem Domain Proteins Create Super Restriction Factors against HIV-1
Source: mBio. 2020 Apr 28;11(2):e00737-20. doi: 10.1128/mBio.00737-20 (PMC7188997; doi:10.1128/mBio.00737-20)
Supplement: FIG S1 [file mBio.00737-20-sf001.pdf]

```
A3C-A3C     MNPQIRNPMKAMYPGTFYFQFKNLWEANDRNETWLCFTVEGIKRRSVVSWKTGVFRNQVD   60
A3F         MKPHFRNTVERMYRDTFSYNFYNRPILSRRNTVWLCYEVKTKG-PSRPRLDARIFRGQVY   59
            *:*::** :: ** .** ::* *    . ** .***: *:     *    .: :**.**

A3C-A3C     SETHCHAERCFLSWFCDDILSPNTKYQVTWYTSWSPCPDCAGEVAEFLARHSNVNLTIFT   120
A3F         SQPEHHAEMCFLSWFCGNQLPAYKCFQITWFVSWTPCPDCVAKLAEFLSEHPNVTLTISA   119
            *: . *** *******.: *   . :*:**:.**:*****..::****:.* **.*** :

A3C-A3C     ARLYYFQYPCYQEGLRSLSQEGVAVEIMDYEDFKYCWENFVYNDNEPFKPWKGLKTNFRL   180
A3F         ARLYYYWERDYRRALCRLSQAGARVKIMDDEEFAYCWENFVYSEGQPFMPWYKFDDNYAF   179
            *****:    *:..*  *** *. *:*** *:* ********.:.:** **  :. *: :

A3C-A3C     LKRRLRESLRNPMKAMYPGTFYFQFKNLWEANDRNETWLCFTVEGIKRRSVVSWKTGVFR   240
A3F         LHRTLKEILRNPMEAMYPHIFYFHFKNLRKAYGRNESWLCFTMEVVKHHSPISWKRGVFR   239
            *:* *:* *****:****  ***:**** :* .***:*****:* :*::* :*** ****

A3C-A3C     NQVDSETHCHAERCFLSWFCDDILSPNTKYQVTWYTSWSPCPDCAGEVAEFLARHSNVNL   300
A3F         NQVDPETHCHAERCFLSWFCDDILSPNTNYEVTWYTSWSPCPECAGEVAEFLARHSNVNL   299
            **** ***********************:*:***********:*****************

A3C-A3C     TIFTARLYYFQYPCYQEGLRSLSQEGVAVEIMDYEDFKYCWENFVYNDNEPFKPWKGLKT   360
A3F         TIFTARLYYFWDTDYQEGLRSLSQEGASVEIMGYKDFKYCWENCVYNDDEPFKPWKGLKY   359
            **********    ************.:****.*:******** ****:**********

A3C-A3C     NFRLLKRRLRESLQLE  376
A3F         NFLFLDSKLQEILE--  373
            ** :*. :*:* *:
```

End of A3C single domain

Linker sequence

S188I S372I A3C polymorphism sites

Conserved A3 cytidine deaminase motif

E68A E254A active site mutation
