# Supplementary figures and images for: APOBEC3C Tandem Domain Proteins Create Super Restriction Factors against HIV-1
Source: mBio. 2020 Apr 28;11(2):e00737-20. doi: 10.1128/mBio.00737-20 (PMC7188997; doi:10.1128/mBio.00737-20)

**A.**

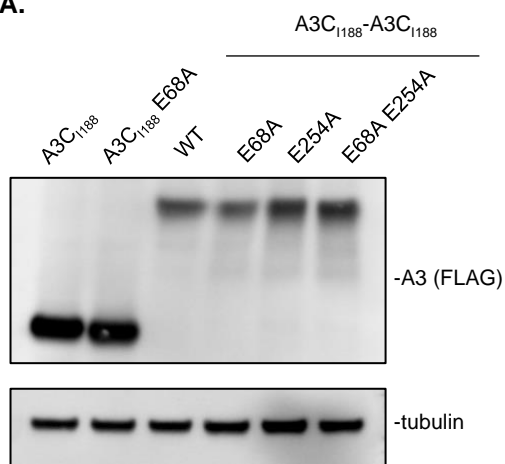

**B.**

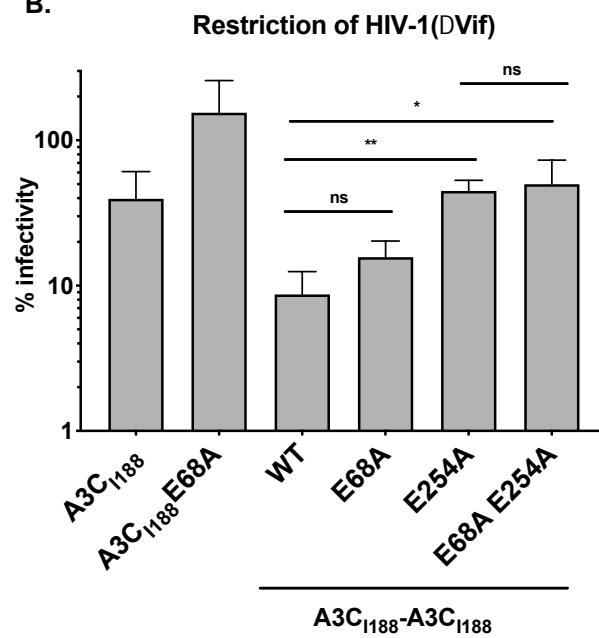

Supplement: FIG S2 [file mBio.00737-20-sf002.pdf]
